# Work values and cultural background: a comparative analysis of work values of Chinese and British engineers in the UK

**DOI:** 10.3389/fpsyg.2023.1144557

**Published:** 2023-05-18

**Authors:** Ziteng Shi, Wei Huang, Yuhui Liang

**Affiliations:** ^1^School of Marxism, Hohai University, Nanjing, China; ^2^School of Labor and Human Resources, Renmin University of China, Beijing, China; ^3^School of Public Administration, Hohai University, Nanjing, Jiangsu, China

**Keywords:** work values, cultural differences, work ethic, habitus, ethnicity

## Abstract

In literature of work-related values and attitudes, it is often argued that different work attitudes could be attributed to different ethnic origins and cultural backgrounds. This perspective article argues that we should avoid a deterministic argument of cultural essentialism and explore how ethnic differences in work values were formed in specifical socioeconomic and cultural contexts. To support this argument, this research conducted in-depth interviews to explore the mechanisms underlying the formation of British and Chinese immigrant engineers’ work values in three dimensions: intrinsic-extrinsic, masculine-feminine, uncertainty avoidance-entrepreneurial risk. The main finding in this paper is that the different social and cultural milieus where both ethnic groups grew up to a large extent contributed to their different work habitus, which further resulted in their different work values. To conclude, this paper contributes to the literature by rejecting the cultural essentialism, which links individual work attitudes with their ethnic/cultural backgrounds in a “deterministic” way.

## Introduction: work values and cultural background

Work-related values and attitudes reflect the degree of importance people place on a variety of job characteristics such as wages, job security and work meaningfulness etc (Qasim et al., 2022; Zhang et al., 2022). In the previous literature, it is often assumed that different work attitudes can be attributed to different ethnic origins and cultural backgrounds (Senhu and Liran, 2021). In the well-known work of Max (2001), it was demonstrated that the Protestant ethics perceived work as a moral obligation and how this facilitated capitalist production. McClelland’s (1961) work argued that the economic development is low in some societies because such societies lack certain cultures for achievement. Also, a study of Claude (1963) on “poverty culture” outlines several cultural characteristics that might lead to poverty such as disintegration into social institutions, hopelessness, despair etc. His research shows that different social groups might have different work attitudes, which could explain their relative success. Moreover, Bourdieu’s (1984) study on French class culture revealed that while people from the working class perceived work as an economic necessity for survival, people from the upper-class regard work as a sense of self-fulfillment.

In addition, Bauder (2003, 2006) conducted several cross-cultural studies to compare the differences between the work attitudes of immigrants and non-immigrants in Canada and examine whether these differences are related to their different ethnic groups, gender, and labor market status. For example, a study of Bauder (2003) indicated that while the experiences of the Communist regime made the Yugoslavian immigrants prefer to enjoy their leisure time with family, the caste system in South Asia (especially in India) tended to make South Asian immigrants to work hard and value social distinction. Another work of Bauder (2006) suggested that people from urban or developed areas were more likely to career-oriented, while those from rural or developing area tended to be more survival-oriented. In addition, Aygün et al.’s (2008) study also supported this view by comparing work ethics between Turkish and American university students. The results indicate that the Turkish students scored higher in Protestant work values than the American students. These cross-cultural studies suggest that the Protestant work values no longer exist only in Western developed countries where Protestant religion is dominant, but can also be found in other cultures, and especially in many developing countries. Their explanations for this argument state that developed countries have now entered a society emphasizing consumption and pleasure rather than the traditional Protestant work values (Aygün et al., 2008).

Religion is also an important dimension of ethnic/cultural backgrounds. Some scholars (Arslan, 2000, 2001; Parboteeah et al., 2009) sought to explore how different religious beliefs affect individual attitudes toward work. Arslan’s (2001) research compared work values of managers from Muslim, Catholic and Protestant cultures. The results indicate that managers from the Muslim culture, for example Turkey, tended to work harder than those from the Catholic and Protestant cultures. The study of Parboteeah et al. (2009) examines the influence of the four major religions in the world including Buddhism, Christianity, Hinduism, and Islam on extrinsic and intrinsic work values. Specifically, they found that people who are affiliated with all religions except Christianity have stronger extrinsic work values than those non-religious people. Similarly, people in all four religions tend to have stronger intrinsic work values.

More recently, a quantitative study (Senhu and Liran, 2021) compares extrinsic and intrinsic work values between the native British and five British ethnic minorities, which are distinguished between first- and second-generation minorities. They found that both first- and second-generation ethnic minorities regardless of specific groups have significantly stronger extrinsic work values than White British, and importantly the ethnic differences become more pronounced for the second-generation groups. Regarding intrinsic work values, first-generation minorities have weaker intrinsic work values compared to White British, whereas the second-generation ethnic minorities have stronger intrinsic work values. The ethnic differences in work values are partly associated with their different demographic and socioeconomic characteristics.

This perspective article argues that we should avoid a deterministic argument of cultural essentialism and explores how ethnic differences in work values are formed in specifical socioeconomic and cultural contexts. The danger of constructing cultural essentialist arguments lies in legitimating the relative success of population groups and justifying the economic disadvantages of others based on their ethnic or racial origins. Such a deterministic notion may also lead policy makers to make value-laden judgments and regard some work-related characteristics of one ethnic group (e.g., hard work) superior to that of other groups (e.g., emphasizing leisure). The main argument in this paper is that the different social and cultural milieus where both ethnic groups grew up to large extent contributed to their different work habitus, which further resulted in their different work values. Thus, this paper contributes to the literature by rejecting the cultural essentialism, which links individual work attitudes with their ethnic/cultural backgrounds in a “deterministic” way.

## Research objective and methods

The overall objective of this study was to explore the formation process of work values of Chinese and British ethnic groups, that is, the socio-economic and cultural contexts in which their distinct work values developed. Particular attention was paid to three dimensions of work values including intrinsic-extrinsic, masculine-feminine, uncertainty avoidance-entrepreneurial risk.

To achieve such objective, semi-structured interview and thematic analysis were used as the data collection and analytic methods. Both methods have been widely regarded as the standard measure to explore the detailed formation mechanisms of social values and attitudes (Bryman, 2008; Babbie, 2020). Specifically, this research conducted a cross-cultural study to explore the patterns of how British engineers and Chinese immigrant engineers in Britain perceived the values and meanings of their work through in-depth interviews of 24 respondents (12 Chinese and 12 British respondents). This study primarily explored three dimensions of work values: intrinsic-extrinsic, masculine-feminine, uncertainty avoidance-entrepreneurial risk (Hofstede, 1980). The two ethnic groups were selected based on the following reasons. On the one hand, China is the largest developing country in the world, which has started its modernization and industrialization process in 1979. While China has its own cultural characteristics, since 1979 there were tremendous changes on people’s values, tastes, and worldviews. This has resulted in a work ethic characterized by industriousness and desires for social distinction in China. On the other hand, Britain was selected as appropriate comparison country because it was the birthplace of modern capitalism and Industrial Revolution at the end of the eighteenth century. As one of the oldest developed countries in the world, Britain represents a distinct set of work values based on the Western cultural system. Such a comparison between Chinese and British ethnic groups could provide valuable insights into our understanding of the social and cultural mechanisms underlying the formation of ethnic differences in work values. For more details about the method and results, see Supplementary material.

## Results and discussion

Overall, this study reveals different mechanisms and patterns for the formation of work values of Chinese and British respondents, which were shaped by the distinct socio-economic and cultural contexts where they grew up. These results hold important implications for a deeper understanding of the relationship between work values and ethnicity.

In the intrinsic-extrinsic dimension of work values, the result highlight the important role of the social milieus where different work habitus of both ethnic groups developed. We found that whilst the economic and social environment where the Chinese respondents grew up has undergone great changes, the environment where the British respondents live is rather stable. Growing up during the process of modernization, Chinese respondents’ desires for better living conditions are unprecedentedly strong and being further strengthened by their hopes to ameliorate the lives of their family members. Such desires have motivated them to work very hard and to focus more on the extrinsic aspects of work. While they moved to another environment, their work habitus has not been completely changed, and still influences their work values and attitudes. In contrast, the British respondents who live in a rather stable period of post-industrialization have far less strong desires for material lives than their Chinese counterparts. Whereas they could be also motivated by personal interest to work hard, such work ethic is less strong compared with the Chinese respondents.

In the masculine-feminine dimension of work values, Chinese and British ethnic groups emphasize both masculine (assertiveness, independency) and feminine (interpersonal connections) dimensions of work values, although the meaning of feminine work values is different for both groups. The patterns of masculine work values are consistent with the results of extrinsic dimension of work values with the Chinese respondents paying slightly more attention to masculine work values such as assertiveness, independency, and emphasis on material lives. This could be explained by the unique socioeconomic contexts they grew up and the modernization thesis (as discussed above). In feminine dimension of work values, both groups emphasize interpersonal relationships. For the Chinese respondents, the interpersonal relationship implies that they must keep good relationships with others from whom they may benefit in the future (Guanxi in Chinese). In contrast, for the British, interpersonal relationship implies that they must well coordinate with their business partners. While interpersonal relationship seems to be an important means to “money” and “achievement,” most British respondents tend to value the intrinsic aspects of cooperation more than the extrinsic. In other words, they tend to embrace cooperation itself more as a valuable quality than as a means. Thus, it seems that the means-end relationship between the masculine and feminine work values is less strong for the British respondents.

In the uncertainty avoidance-entrepreneurial risk dimension of work values, we found that the British respondents are more willing to take entrepreneurial risks and devote more efforts to entrepreneurial activities, which was driven by their personal interest, self-actualization as well as pursuit for better material lives. It is the structural barriers, especially cultural barriers which had largely prevented many of our Chinese respondents from engaging in some entrepreneurial activities. They enjoy their stable lives, prefer business with low cost and try to avoid high entrepreneurial risks. In contrast, the British respondents who are residents in Britain naturally enjoy more entrepreneurial advantages over the Chinese respondents such as social network. Thus, compared with the Chinese respondents, the British respondents seem to emphasize individual initiatives and entrepreneurial risks to a larger extent.

Overall, these results echo our argument that we should avoid a deterministic argument of cultural essentialism and explores how ethnic differences in work values are formed in specifical socioeconomic and cultural contexts. The danger of constructing cultural essentialist arguments has adverse political and economic consequences. It may not only lead to stigmatization of certain ethnic groups in socioeconomic life chances, but also could result in misleading and value-laden policies which legitimate the relative success of population groups and justify the economic hardship of others based on their ethnic or racial origins. However, our results clearly show that the different social and cultural milieus where both ethnic groups grew up to large extent contributed to their different work habitus, which further resulted in their different work values. Thus, this paper contributes to the literature by rejecting the cultural essentialism, which links individual work attitudes with their ethnic/cultural backgrounds in a “deterministic” way.

## Data availability statement

The original contributions presented in this study are included in the article/Supplementary material, further inquiries can be directed to the corresponding authors.

## Ethics statement

Ethical approval was obtained from the corresponding institution to conduct this research.

## Author contributions

ZS carried out the research and the manuscript writing. WH and YL contributed to the theoretical framing and writing during the revision. All authors contributed to the article and approved the submitted version.
