# Supplementary material for: Work values and cultural background: a comparative analysis of work values of Chinese and British engineers in the UK
Source: Front Psychol. 2023 May 18;14:1144557. doi: 10.3389/fpsyg.2023.1144557 (PMC10232762; doi:10.3389/fpsyg.2023.1144557)
Supplement: Supplementary file 1 [file Presentation_1.pdf]

Online Supplementary Materials

Methods

This research was based on the information collected from in-depth interviews. In this research, our interviews were divided into two kinds. First, we conducted some informal interviews which were unstructured and unrecorded for several purposes. These informal interviews not only significantly helped us familiarize with the background knowledge, but also helped establish formal interview questions. During this study, we interviewed some people who should not be included in the samples but are crucial for the understanding of Chinese work values. For example, when we found that the work values of Chinese respondents were greatly impacted by their parents, we decided to interview some old Chinese engineers whose ages are like the respondents' parent generations. By interviewing them, we have gained a better insight into how the work values of Chinese respondents were impacted by their parent generations. Second, by following the formal questions, we employed semi-structured interview, which roughly followed a set of questions while at the same time allowing spontaneous interactions between interviewer and respondents (Bryman, 2008). All formal interviews were recorded, and most data of this research were based on these interviews. Semi-structural interview is appropriate in this study as it could not only examine the three dimensions of work values but is also able to reveal detailed patterns in each dimension (Bryman, 2008).

Since our study is explorative in its nature, we employed a purposive sampling method. This sampling method was based on the substantive knowledge of population and purpose of this study, thus being non-representative of the whole population (Bryman, 2008). As this study focuses on cross-cultural comparisons of work values between Chinese and British ethnic groups, we studied engineers' values and attitudes towards their work for two reasons. First, the content of engineers' work is primarily about techniques and is also similar across different cultures, which may facilitate our cross-cultural comparison. In contrast, some occupations such as doctor may be unsuitable in this research. For example, the work content of traditional Chinese doctors and British doctors is essentially different, which may make both groups less comparable in terms of their work values and attitudes. Second, in the occupation of engineering, there are very clear boundaries between normal work and extra work, work time and non-work time. This may also help compare different ethnic groups' attitudes towards work and leisure. Contrarily, some occupations such as policemen are also not suitable for this research because their work time is relatively unfixed compared with engineers. It is sometimes not very clear whether they work or not, and how much they have worked, which makes these occupations less comparable across cultures.

The respondents were also recruited by following a purposive sampling method. We have chosen 12 Chinese immigrant and 12 British engineers to conduct interviews. The British engineers are all British citizens who were born in Britain and have received education here. Their ages are between 35-46 years old, and all of them received university education (5 under-graduates and 7 post-graduates). The Chinese immigrant engineers were all born in China and moved to Britain and then acquired the British citizenship. Their ages are between 38-45 years old and almost all of them received post-graduate education either in China or aboard (1 under-graduate and 11 post-graduates). Thus, since these Chinese immigrant engineers are now British citizens, they enjoy the same governmental policies and economic institutions as the British engineers do. However, as they were educated and lived primarily in China, their values and worldviews should be to some extent distinct from their British counterparts. Furthermore, the cohort of Chinese engineers experienced the tremendous

social changes in Chinese society. In such a social milieu, they might have developed a distinct work *habitus* including all work-related values, attitudes, and behaviors. Comparing them with their British counterparts living in a society where the dominant social values are rather stable may reveal informative results.

The sample size was not determined before interviews. Instead, several samples were drawn from both ethnic groups until theoretical saturation was achieved (Glaser and Strauss, 1967). For example, if the responses of several participants did not provide satisfactory information to the research question, we draw more participants and arrange more interviews. Or if it was found that additional interviews no longer provided any new information and data to the research question, we stopped interviewing more participants. Or if new topics emerge during the interviews, we also modified the sample size by adding more participants. Overall, the method of sampling is in line with the explorative nature of this research.

The formal interviews were then transcribed, coded. We employed an inductive coding, which is a method primarily driven by data and aims to reveal some patterns from data. The codes were analyzed and categorized into several main themes. Then, we employed thematic analysis, which is defined by Braun and Clarke (2006) as a flexible method for exploring patterns within the data and capturing some crucial detail regarding the research question. Generally, there is a distinction between semantic and latent themes. The former refers to the explicit meanings of data, while the latter refers to the implicit and underlying meanings (Braun and Clarke, 2006). In this study, both kinds of themes are important because the purpose of this study is to explore the respondents' work values as natural as possible. In some cases, the explicit meanings of data can reflect the real feelings and thoughts of the respondents. This requires the interviewer to be very sensitive about the respondents' expressions and behaviors. Having identified the themes, we then uncovered the mechanisms underlying the formation of ethnic differences in work values.

Results

Intrinsic vs Extrinsic

In this dimension, we examined the extent to which the work values of both groups are intrinsic or extrinsic by exploring their work motivations and attitudes towards Protestant work values. And through detailed exploration of the interview transcripts of both ethnic groups, we also found that the mechanisms through which both groups rationalized their work motivations are very different. We found two main themes of the Chinese group "money" and "family". Under "family", there are two subthemes "parental influence" and "devotion". For almost all Chinese respondents in this study, industriousness remained a significant value that has been socialized into them in their early education. The primary motivation behind their hard work is to earn much money in order that their family members especially their children and parents could have better lives in the future. Under the "family" theme, "parental influence" remains an important subtheme, which could possibly illustrate why they emphasize industriousness and diligence. As the Chinese respondents said:

"My parent generations were born in 1950s that was the Communism time of China. At that time especially during the Great Leap, diligence and frugality were greatly propagated by the Chinese government and regarded as the most important social virtues, which every Chinese citizen should strive for. It was normal at that time to work extra hours, and people also felt

honored and pleased to work hard as they believe that every little thing they did would finally contribute to the establishment of Communism society."

In this quote, it seemed that for the parent generations of our respondents, their work motivations were more intrinsic rather than extrinsic. In other words, they regarded the work itself as an honored activity rather than a means to earn money. And such impacts seem to be particularly evident, when I found that the Chinese respondents now live in a society (i.e., Britain) where people's attitudes towards leisure are much more tolerant than that in China. As one Chinese respondent said:

"When I just worked in Britain, I was the only person in my company who would like to go to the office on Sunday. ... But I gradually found that my British colleagues never did extra works after normal work time, and they often told me that I only need to finish the required tasks, and then all the rest of time is free. ... So I think my work attitudes have been changed after the immigration to Britain, ... for example, I tend to spend more time in leisure activities and with my families. However, we are still very different from the British local residents. They never did extra work, but for me, of course no one likes doing extra work, if my required tasks are not finished, I would feel obliged to finish them even in my leisure time."

Whilst the parent generations of the respondents emphasized the intrinsic value of work, all respondents regard the primary purpose of work to earn money. This difference could be possibly attributed to the different social milieu where they lived. As one respondent described:

"In the time of my parents, people's desire for money was not very strong. Due to the scarcity of commodities, rich people actually lived a similar life as poor people did. Thus at that time money did not mean much to us. However, since the implementation of free market policies in 1970-80, everyone has chances to earn much money and improve his or her life. ... Nowadays houses, cars, children's education etc. everything needs money. ... Therefore, my main work motivation is also to earn money and provide good lives for my family members."

From this quote, we found that these social changes make Chinese people's desire for better life and social distinction unprecedentedly strong. It seems that since then, their work values have changed from intrinsic to extrinsic. Their hard working is no longer to practice certain beliefs or to achieve a sense of self-actualization. A stronger driving force motivates them to work hard, that is, money. They make money to return the raising of their parents and save up for their children. Thus, the second subtheme under "family" is "sacrifice" or "devotion". While the respondents also mentioned that personal interests remain an important reason for why they chose this occupation, "parental influence" and "devotion" may illustrate the main mechanism that why Chinese immigrant engineers tend to work hard and focus on extrinsic aspects of work. On the contrary, we found that "money" and "personal interest" are the main themes for the British group. Like the Chinese, most British respondents also regard money as an important motivating them to work. As several British respondents said:

"I really think money is very important, especially if you want a comfortable life. Money is necessary to maintain even the most modest lifestyle. It can be argued that everyone needs money, if you do not want to depend on others."

However, compared with the Chinese, they pay less attention to the extrinsic value of work than the Chinese respondents do. While most of the British respondents attach significant importance to the material return of their work, a similar proportion clearly expressed that personal interest should be the most important determinant of one's occupation and career. As two British respondents said:

"I think it is my interest in this that makes me want to do this job, and also a desire to make a comfortable environment where people could work and live in. I don't know if this applies to others, but this is the case for me. ... And finding solutions to problems always excites me. That is an awesome feeling!"

"The most important thing is to take the time to get to know yourself: what kind of things you are willing to do, what really makes you happy, or what really interests you. And also get to know what sort of environment you like to work in."

Thus, for British respondents both personal interest and material rewards drive their industriousness. The former reflects the intrinsic value, and the latter reflects the extrinsic value of work, and both remain almost equally important. Overall compared with the Chinese, they seem to place less emphasis on the extrinsic aspects of work. This is especially evident in some cases in which they would rather pursue their own interests than high salaries. As one British respondent mentioned:

"After eight years of working for others, I decided to do what really interests me. My business partner and I established a company, designing for individual clients, by selling some self-made art, crafts and furniture. But for three years, there were only two full-time staff in the company, namely me and my partner, as the profit made was too little to hire another full-time employee. Despite this, I still enjoy what I am doing. ... Due to the small number of employees I have almost no free time with my family until we hire some student interns to take on some of the workload."

In this quote, we found while the respondent earns less money now than he did, he still devotes much time to his current company and very enjoys his current work. It is his interest rather than money that drives him to do so. This kind of experience could not be found among the Chinese respondents. For the Chinese respondents, it is both family and material rewards that mainly motivate them to work hard. However, both motivations reflect the extrinsic values of work. Thus, the overall work motivations of Chinese respondents tend to be more extrinsic than their British counterparts. In terms of the work attitudes, like the Chinese, the British respondents equally emphasize industriousness. During the interviews, I found that all of them regard hard work as an important work value and necessary especially in their occupation.

"Design is an ongoing occupation that never comes to a stop and involves constant research to new technology and innovative fashions. You must work very hard to keep up with new artistic and fashion development. ... Also, all the great designers are self-educators and travellers. They must go to a lot of places all over the world so they can draw inspiration from different cultures."

However, in their minds there should be a clear boundary between work and non-work (leisure) time. Most British respondents clearly expressed that they prefer to work hard in normal work

time and fully relax in their leisure time. While many of them to do not strongly refuse to do some work-related things in free time, they still need enough leisure time to relax.

"Outside of work, I am also willing to do some work-related things, such as learning to design software, or visiting a fashion exhibition. But I think I really need a certain amount of free time away from work to fully relax and possibly pursue other hobbies."

"During holidays, if you try to work, even just for one or two hours, I think you will feel a great sense of accomplishment. But breaking is also important. If you are not giving time to relax and enjoy your life, for what reasons you are working."

To explore the social milieus that developed different work *habitus* of both ethnic groups, we found that whilst the economic and social environment where the Chinese respondents grew up has undergone great changes, the environment where the British respondents live is rather stable. Grew up during the process of modernization, Chinese respondents' desires for better living conditions are unprecedentedly strong and being further strengthened by their hopes to ameliorate the lives of their family members. Such desires have motivated them to work very hard and to focus more on the extrinsic aspects of work. While they moved to another environment, their work *habitus* has not been completely changed, and still influences their work values and attitudes. In contrast, the British respondents who live in a rather stable period of post-industrialization have far less strong desires for material lives than their Chinese counterparts. Whereas they could be also motivated by personal interest to work hard, such work ethic is less strong compared with the Chinese respondents.

Masculinity vs Femininity

In this dimension, we explored the extent to which both ethnic groups emphasize the masculine and feminine work values. Throughout the interviews, we found that both groups paid almost equal attention to both work values, although the Chinese respondents emphasized both work values to a greater extent. In terms of the masculine work value, this result is consistent with what we have concluded in previous section that the Chinese respondents focus more on the extrinsic work values and their desires for better material lives are stronger than their British counterparts. The masculine values are defined as work values emphasizing achievement, assertiveness and acquisition of material rewards. It is then not surprising that the Chinese respondents are aligned with the masculine work values to a greater extent than the British respondents. In addition, this result is also consistent with the current literature arguing that people who are from developing area and have experienced the modernization process are more likely to emphasize money, social distinction; whilst people who are from developed area and live in a post-capitalistic society tend to value family and interpersonal relationships (Niles, 1994; Arslan, 2000).

While roughly consistent with the current literature, the results in this research reveal more detailed patterns, which might have been overlooked by the literature. In terms of the Chinese respondents, we found that "money" and "interpersonal connections" are two main themes. The former represents the masculine value, and the latter reflects the feminine value. As mentioned previously, Chinese people who experienced the free market reform since 1978 tend to value the masculine work values, for example, assertiveness, independency, and emphasis on material lives (Feather, 1998; De Mooij, 1998). However, this does not imply that the traditional Chinese work culture has been completely abandoned. In contrast, they still attach great importance to the feminine work values especially interpersonal relationships. Since we

have already explained the reasons that the Chinese respondents emphasize material rewards of work, in this section we paid particular attention to the feminine work values of the Chinese respondents. Here we quoted one Chinese respondent to show his emphasis on the feminine work values:

"Chinese people usually attach great importance to guanxi (social networks). In China, it works in almost all circumstances regardless of you want to find a job, get to school or start a business. This is especially so in workplace, keeping a good relationship with your superiors may probably help you get more promotion chances. ... Actually no matter within or outside workplace, maintaining good relationships with people around you is a kind of investment, which may benefit you in the future. ... When I worked in China, to obtain a promotion chance I even helped with my superior's son with his homework, which seems unbelievable in Britain."

This quote to a large extent represents the typical opinions of the Chinese respondents who place a great emphasis on *guanxi*. Although the extent to which Chinese people emphasize *guanxi* may vary according to different regions where they come from, all Chinese respondents have mentioned *guanxi* in the interviews. What they called *guanxi* can be divided into two kinds: the relationship with their work colleagues, and the relationship with their superiors. The latter was particularly emphasized. Furthermore, the quote also indicates that their emphasis on *guanxi* could be attributed to their work *habitus* developed in the Chinese work environment. More importantly, the Chinese respondents' emphasis on *guanxi* and hierarchical relationship is only limited to the workplace but extends to the rest of their lives. However, after their immigration to Britain they seem to pay less attention to establishing *guanxi* than they did in China. As one Chinese respondent mentioned:

"Gunaxi is also important for British people, but I think the work atmosphere in Britain is much better than that in China. Here one's promotion primarily relies on his or her own strength, and people do not need to always consider about how to invest in personal connections and how they could benefit from them. This is also a reason why I chose to stay here. ... But guanxi as such is not a bad thing. Particularly for immigrants like us in Britain, it is very necessary to establish a close friend circle and my network of guanxi because guanxi is important for our immigrants to find feet in a new country. ... I also often tell my children about how to establish their guanxi either in schools or in society."

As showed in the quote, after moving to Britain, many Chinese respondents said that while British people also emphasize *guanxi*, they pay much less attention to it than Chinese people. Thus, they enjoy the work atmosphere in Britain. Nevertheless, as they said, *guanxi* as such is not harmful. If possible, they still would like to pay particular attention to establishing their *guanxi* networks. In addition, we found that the Chinese respondents' feminine work values (especially *guanxi*) do not exist alone but are closely associated with their masculine work values. It seems that there is a means-end relationship between both work values. In other words, the Chinese respondents pay much attention to *guanxi* to take advantage of such connections to acquire money, promotions or achieve social status, which could probably help them find their feet in Britain.

In terms of the British respondents, we found that "money" and "achievement" are the main themes, which represent the masculine work values. Compare with the Chinese respondents who only have one theme "money", the British respondents seem to pay equal attention to both material lives and individual self-actualization. This result further confirms our conclusion in

the previous section that the British respondents' work motivations are closer to the intrinsic end in the intrinsic-extrinsic continuum compared with their Chinese counterparts. For the feminine work values, we found one main theme "interpersonal relationship" and one subtheme "teamwork" or "cooperation". However, for the British respondents, interpersonal relationship does not refer to a special meaning as *guanxi* implies to the Chinese people. Instead, their understanding of interpersonal relationship is more general and comprehensive, including friends, work colleagues, business partners, and of course *guanxi*. Of particular importance, they have mentioned many times that their occupation requires one's capacity to well communicate and cooperate with others. For example, several British respondents who are interior decoration designers mentioned the importance of cooperation in decorating a new house. As they said:

"My daily routine is varied. ... You need to learn the basics before you can even pick up a pencil. The tasks include designing a piece of furniture, project coordination, client or supplier liaison, meeting clients, and visiting exhibitions. As a whole, I would say I spend more time in coordinating than designing, and I know that without the organization, my designs can never be realized."

"I think design is an occupation that requires very good communication skills. It is very important that a designer is competent enough to translate his or her ideas on to paper through drawings, and that he is capable of instructing and managing craftsmen and builders in construction teams."

From these quotes, the British respondents emphasize different aspects of feminine work values compared with the Chinese respondents. For the Chinese respondents, the interpersonal relationship implies that they must keep good relationships with others from whom they may benefit in the future. In contrast, for the British, interpersonal relationship implies that they must well coordinate with their business partners. For example, as an interior designer, one must firstly be clear about what customers want, for instance, the design style, the materials. He then must accurately translate the ideas and requirements of customers into design drawings. All these designs must be finally realized by a construction team. While "cooperation" seems to be an important means to "money" and "achievement", most British respondents tend to value the intrinsic aspects of cooperation more than the extrinsic. As they said, it is their interests to "make beautiful environments for people to live and work in. Finding the solution that you have been looking for or struggling to find is an awesome feeling". In other words, they tend to embrace cooperation itself more as a valuable quality than as a means. Thus, it seems that the means-end relationship between the masculine and feminine work values is less strong for the British respondents.

Uncertainty Avoidance vs Entrepreneurial Risks

In this dimension, we examined the extent to which both ethnic groups tend to avoid uncertainty or are more willing to take entrepreneurial risks. As explained, British dominant culture is characterized by encouraging individual initiatives and entrepreneurial activities, whereas the Chinese traditional culture is characterized by emphasizing stability and collective decisions. However, recent social transformations since 1978 have greatly changed Chinese people's entrepreneurship orientation. In response to such social changes, there emerged many entrepreneurs in China (Yang, 2007). It is argued that Chinese people's enthusiasm for entrepreneurship was unprecedentedly strong even if relevant economic institutions were still

incomplete. We have thus assumed that the Chinese respondents are more likely to show a positive attitude towards entrepreneurial activities than the British respondents.

By analysing the interviews, we found that the results revealed in this section are reverse to what we have assumed. In other words, the British respondents are more willing to take entrepreneurial risks and devote more efforts to entrepreneurial activities. During the interviews, we found that "family" again is the main theme and "stability" is the subtheme for the Chinese respondents. We also found that the reason why these Chinese respondents are less willing to engage in entrepreneurship might not be attributed to their work values developed in China. Instead, the reason could be that these Chinese immigrants have encountered cultural barriers. Almost all Chinese respondents have mentioned that they would prefer to do business with Chinese in Britain and earn money from them. As several Chinese respondents said:

"I would like to do business with Chinese in Britain. To do this, I could bypass some tedious routines, which I must perform when dealing with British people. ... Apart from this, I think there are still great cultural differences between Chinese and British people. Whilst I have lived in Britain for over ten years, I think I still did not integrate into British society."

"I prefer to do some part-time business with Chinese rather than British people. With more and more Chinese investing in British housing estates, I can not only help them design their houses, but also help them contact with real estates developer, and even borrow mortgage loans from banks. While my business has gone well, I am not very willing to start a real career such as establishing a company. A primary reason is that I am only acquainted with designing techniques and is reluctant to spend a lot of time to familiarize with British laws and regulations. In addition, starting a real business is very risky. I do not want to take such a big risk unless there was an excellent opportunity."

Their opinions reflect the work values of most our Chinese respondents who are unwilling to take many risks and prefer a stable life in Britain. While they often do some business, which can be defined as entrepreneurial activities, the population they deal with is primarily limited to the Chinese. More importantly, they seem to only prefer business with small cost and low risks. As one Chinese respondent explained:

"I arrived in Britain in early 1990s; at that time there were fewer Chinese than today. I did not know how to deal with government officers and local business partners. My wife and I even spoke English with strong Chinese accent, let alone starting a business. ... I prefer a stable life, because if my business had failed, my family members' lives especially my children's lives might be influenced."

In this quote, it seems that the respondent has never thought that he could become a real entrepreneur who is able to utilize available resources. Thus, for these Chinese respondents, doing small business with Chinese in Britain may be the "safest" entrepreneurial activity, and the "safest" way to improve the material conditions of their family member's lives. These Chinese respondents' attitudes towards entrepreneurial risks are sharply contrasted with their British counterparts. Throughout the interviews, we found that the British respondents appreciated entrepreneurial risks to a larger extent than the Chinese respondents. We also found that "personal interest" becomes the main theme, which possibly motivates them to be willing to take entrepreneurial risks. As a British respondent mentioned:

"I began to save money during my previous job to prepare for starting up this company. However, after three years, the company is still during the start-up stage, and I am uncertain about the company's future. Although I do not think that the decision carries a high risk. In addition, I also do some part time work in schools and universities, so even if the company possibly fails in the future, I am still confident that I can sustain a modest life for my family. Most importantly, I am doing what really interests me and is something that I am passionate about."

Whilst not all British respondents are now entrepreneurs, most of them clearly expressed that they do not exclude the possibility of taking some entrepreneurial risks and engaging in entrepreneurial activities one day in the future. Moreover, they did not regard entrepreneurship as an inherent quality, rather a skill, which can be learned through practice. When engaging in entrepreneurial business, the British respondents certainly have advantages over their Chinese counterparts because they have more and easier access to economic resources and social capital. It seems that the difference in the extent of taking entrepreneurial risks between both groups could not be attributed to their different work *habitus*, but to some structural barriers that the Chinese respondents have encountered when they arrived in Britain. However, as argued, work *habitus* is not invariable, but may also change in response to environmental changes. In this study, for example, whilst the Chinese respondents were born in the time of Chinese entrepreneurial zeal, after their immigration to Britain, the structural barriers seemed to prevent them from engaging in many entrepreneurial activities which they could do very easily in China.

In such an environment, they seemed to emphasize stable lives more than entrepreneurial risks, individual careers or achievements. As explained in the intrinsic-extrinsic section, the Chinese respondents have paid increasing attention to leisure, being with their family and other non-work activities after their immigration to Britain. These changes in their work values are also consistent with their emphasis on stable lives. The stable lives in their minds should and must be achieved by hard working. Therefore, as observed from our Chinese respondents, they worked very hard, but they did not have great ambitions and only wanted stable lives. In other words, they have developed their distinct work *habitus* which might be different from that of their counterparts in China. As one Chinese respondent mentioned:

"*Many of my university classmates started their entrepreneurship after graduation, and now they become millionaires or even billionaires. However, I do not envy them. ... I feel very satisfied with my current life, because I can spend the time that my millionaire friends devoted to their careers with my families."*

To sum up, the findings in this section reveal that the British respondents seem to emphasize individual initiatives and entrepreneurial risks more than the Chinese respondents. This seems to be contrary to our expectation that the Chinese respondents who have undergone China's the modernization process are more likely to be willing to accept and tolerate entrepreneurial risks than the British respondents who live in a post-industrial society. Throughout the interviews, we found that it is the structural barriers, especially cultural barriers which had largely prevented many of our Chinese respondents from engaging in some entrepreneurial activities. As a result, our Chinese respondents seemed to develop a distinct work *habitus*, which is to some extent characterized by post-industrialization. They enjoy their stable lives, prefer business with low cost and try to avoid high entrepreneurial risks. In contrast, the British respondents who are residents in Britain naturally enjoy more entrepreneurial advantages over

the Chinese respondents. Thus, compared with the Chinese respondents, the British respondents seem to emphasize individual initiatives and entrepreneurial risks to a larger extent.
